# Emerging Topics in Cardiometabolic and Psychologic Sequelae, Pathogenesis, and Treatment of Polycystic Ovarian Syndrome: A Review

**DOI:** 10.3390/children6080089

**Published:** 2019-07-31

**Authors:** Rachana Shah

**Affiliations:** Division of Pediatric Endocrinology, Children’s Hospital of Philadelphia and Perelman School of Medicine at the University of Pennsylvania, 3615 Civic Center Blvd, 802F Philadelphia, PA 19104, USA; shahr@email.chop.edu

**Keywords:** obesity, fertility, metabolism, gynecology, ovary

## Abstract

Polycystic ovarian syndrome (PCOS) is a common endocrine disorder, affecting 6–10% of reproductive age women and influences the reproductive, metabolic, dermatologic, and psychiatric health of affected girls and women. Despite its prevalence, the pathogenesis of the disease is largely unknown, and treatment options are limited. Thus, PCOS has been a ripe area for research in recent years, and novel etiologic pathways, diagnostic parameters, and treatment options are being explored. This review focuses on recent data suggesting pathogenic and therapeutic considerations, as well as the psychiatric and metabolic sequelae of PCOS.

## 1. Introduction

Polycystic ovarian syndrome (PCOS) is the most common endocrine disease in reproductive age women, with reported prevalence of 6–10% in the general population. PCOS features a combination of anovulation and hyperandrogenism, which clinically present as irregular menstrual periods and infertility, as well as hirsutism and acne. Increased awareness and screening have led to increasing diagnosis in the adolescent population. While insulin resistance, impaired gonadotropin signaling, and altered ovarian reactivity have all been implicated in the pathogenesis, the primary underlying cause of PCOS remains unknown [1]. Thus, management of the disorder is primarily focused on alleviating symptoms. Fortunately, there has been significant progress in clarifying diagnosis and management, elucidating the pathogenesis, and developing therapeutics to help the millions of women worldwide suffering from PCOS. 

This review focused on providing a summary of recent advances in clarifying the comorbidities, underlying pathogenic pathways, therapeutic options, and long-term sequelae of PCOS.

## 2. Diagnosis and Management of PCOS 

Symptoms of PCOS often emerge during adolescence, but diagnosis can occur at any time during the reproductive years. There are three commonly utilized diagnostic criteria for PCOS in adult women, all of which begin with ruling out other etiologies of symptoms. In the Rotterdam criteria, patients must meet two of the following three: clinical or biochemical hyperandrogenism (acne, hirsutism, or alopecia and/or elevated free or total testosterone), oligo-ovulation or anovulation (>35-day cycle or midluteal progesterone <2.5 ng/mL), and polycystic ovaries (>10 mL volume, or >12 2–9 mm follicles in at least one ovary). The National Institute of Health (NIH) criteria require *both* hyperandrogenism and menstrual dysfunction and the Androgen Excess and PCOS Society (AE-PCOS) guidelines require hyperandrogenism plus ovarian dysfunction and or polycystic ovaries (with follicle count cutoff raised to 25 due to more sensitive current imaging techniques) [2].

Recent consensus guidelines have established diagnostic criteria for adolescent PCOS to require the combination of the following in girls at least two years from menarche: (1) oligo- or anovulation (defined as <8 cycles per year or cycles <21 or >35 days in length or amenorrhea for >90 days for any one cycle or primary amenorrhea by age 15 or >3 years post thelarche); (2) hyperandrogenism, both biochemical (elevated free or bioavailable testosterone levels by sensitive assay) or clinical (hirsutism, severe acne not responsive to topical treatment) [3]. Ultrasound criteria are not utilized in adolescents due to lack of age-specific normative data and cut-off values. The adolescent guidelines for making a diagnosis of PCOS are more specific but potentially less sensitive, thus requiring careful follow up over time in girls that do not fully meet criteria at initial evaluation [4,5,6]. 

Once the diagnosis is established, management of PCOS should include: (1) regulation of menstrual cycles (and restoration of ovulation if fertility is desired); (2) assessment and treatment of dermatologic concerns (hirsutism, acne, androgenic alopecia) with cosmetic or pharmaceutical options; (3) screening for cardiometabolic comorbidities (e.g., insulin resistance, obstructive sleep apnea, hypertension) and counseling on lifestyle modification—calorie reduction and increased physical activity—to improve weight and metabolic parameters; and (4) screening and counseling for mental health conditions and appropriate referrals for mental health care [7].

## 3. Beyond Reproduction: Current Understanding of Comorbidities in PCOS

While irregular menses, infertility, acne, and hirsutism can majorly impact health and quality of life (QOL)in girls with PCOS, the risks of PCOS extend well beyond the readily assessed gynecologic and dermatologic symptoms and can have wide-ranging impacts on a women’s physical and mental health throughout her life. 

### 3.1. Cardiometabolic Risk

PCOS is associated with significant cardiometabolic which begins to manifest in adolescence [8]. Adolescents with PCOS are more likely than age- and body mass index (BMI)-matched controls to have insulin resistance [9], dyslipidemia, and non-alcoholic fatty liver disease. Akgul et al. recently reported that 52% of adolescent girls with PCOS in their cohort met criteria for metabolic syndrome [10], which is well above the rate reported in obese non-PCOS teens. A meta-analysis of seven studies confirmed these findings, reporting odds of metabolic syndrome in PCOS as 2.69 times higher than girls without PCOS [11].

Looking beyond traditional metabolic syndrome parameters, complex determinants of cardiometabolic risk are also higher in PCOS teens than age and BMI-matched control girls. Increased carotid intima thickness (CIMT), beta thickness, and reduced arterial compliance, as well as atherogenic lipid profile, in PCOS compared to BMI-matched controls have been reported by one group [9]; however, another study showed similar rates in obese PCOS and obese-non PCOS subjects of CIMT, pulse wave velocity, and inflammatory markers [12]. Using indirect calorimetry and hyperinsulinemic-euglycemic clamps, Kim et al. determined that girls with PCOS had decreased lipid mobilization and fat oxidation, and metabolic inflexibility as a phenotype of adipose tissue dysfunction, despite similar BMI and body fat percentage as a control group [13].

Several novel biomarkers associated with adiposity and metabolic derangements have been assessed in PCOS. Irisin is a myokine expressed in muscle and adipose tissue, induced by exercise, and leads to adipose tissue browning, increased energy expenditure, and improvements in glucose homeostasis in a mouse model [14]. Its benefits on glucose tolerance has been related to increased glucose transport and decreased hepatic gluconeogenesis in an adenosine monophosphate-activated protein kinase (AMPK) pathway [15]. In PCOS, circulating levels of irisin are elevated and associated with metabolic, androgenic, and reproductive markers [16,17], with one small trial showing reduced levels with metformin treatment [18]. Conversely, levels are reduced in subjects with type 2 diabetes and gestational diabetes [19], suggesting a regulatory role that is induced by obesity and insulin resistance and compromised with beta-cell failure and further metabolic derangement.

The adipokine S100A4 has been implicated in subcutaneous adipose tissue dysfunction and insulin resistance [20]. In mice, S100A4 levels were reduced after high fat diet and S1004 deficient mice had reduced insulin signaling, increased obesity, and liver inflammation [21]. In girls with PCOS, S100A4 levels were elevated and correlated with hepato-visceral adiposity; S100A4 and hepato-visceral fat decreased further with a combination spironolactone/pioglitazone/metformin (SPIOMET) treatment than with oral contraceptives [22].

Fetuin, a glycoprotein expressed predominantly in liver and adipose tissue, has been broadly associated with obesity and cardiometabolic dysfunction [23]. In human cell lines, fetuin A blocked insulin receptor tyrosine kinase activity which is critical for insulin signaling [24]. Levels of fetuin A are higher in type 2 diabetes [25] and metabolic syndrome, and levels reduced with weight loss [26], exercise [27], and metformin [28]. Results on associations of fetuin A to PCOS have been conflicting. Several studies have reported found levels were higher compared to age and BMI-matched controls [29,30] and positively correlated with insulin, HOMA-IR, and free androgen index [31], while another found no difference [32]. In a small adolescent study, fetuin A levels were lower in PCOS and normalized with SPIOMET [33,34]. The role of fetuin in PCOS and its relationship to hormonal and metabolic markers requires further study.

The insulin-sensitizing adipokine omentin-1 is secreted by visceral adipose tissue and increases glucose-stimulated insulin secretion in adipocytes [35]. Omentin expression and secretion in adipose is down-regulated by glucose and insulin ex vivo, and circulating levels reduced by inducing hyperinsulinemia in healthy subjects [36]. Lower levels are seen in obese but otherwise healthy children [37], suggesting a role early in metabolic derangement. In PCOS, levels are reported to be reduced in both lean and obese subjects [38], with levels correlating negatively with hyperandrogenism [39] and increased after treatment with both estrogen-progestin oral contraceptives (OCP) [40] and metformin [41].

### 3.2. Psychiatric Co-Morbidities

In the past several years, the high rate of mood disorders in women with PCOS has come to light. Adult studies have shown increased rates of anxiety and depression when matched for BMI [42,43,44], though data in teens remains very limited. An internet-based survey conducted in Germany found that maladaptive coping behavior associated with depression and anxiety symptoms [44]. Another group reported that reduced health related QOL was predicted by self-esteem, body image, and sexual function in a cohort of women with PCOS in Iran [45] similar to a study in Poland reporting low rates of self-attractiveness that correlated with poor sexual function [46]. A small qualitative study revealed that girls with PCOS perceived lack of control, and were most concerned about menstrual issues and fertility prognosis, with lower levels of control predicting depression [47].

Notably, there appears to be a relationship between insulin resistance and depression [48,49]. In a recent pilot study of adolescent and adult PCOS, Erensoy et al. demonstrated that treatment with metformin for 90 days led to reduction in depression and anxiety scores, along with weight and insulin resistance [50], though mechanism of how metformin effects mental health—directly or indirectly—are unknown. Benefits of lifestyle modification on mental health in PCOS have also been demonstrated. Physical activity itself has been shown to decrease depression and increase QOL in PCOS [51,52]. Dokras et al. reported that both weight loss via weight loss and oral contraceptive pill use to treat hyperandrogenism led to improved depression, anxiety and QOL scores, with additive benefits in the combined treatment group [53]. Addressing psychological concerns with cognitive behavioral therapy improved QOL but also weight loss during a lifestyle intervention program in overweight or obese PCOS women highlighting the importance of identifying and treating mental in addition to physical health concerns [54].

A study evaluating efficacy of an 8-week a mindfulness program compared to placebo showed that the treatment led to improvements in mental health parameters and reduction in salivary cortisol in PCOS women [55], suggesting that multiple modalities of therapies should be explored.

## 4. Setting the Foundation: Proposed Pathogenic Mechanisms

Despite its prevalence, the pathogenesis of PCOS remains incompletely understood. Alterations in ovarian structure and function, adrenal steroidogenesis, pituitary gonadotropin pathways, insulin signaling, gut microbiome, environmental exposures, and genetic and epigenetic markers have all been implicated and are areas of current research focus.

### 4.1. Fetal Origins

Origins of ovarian dysfunction, including PCOS, likely begin early in fetal development. Germ cells migrate to the gonadal ridge and undergo mitosis until around 20 weeks of gestation, reaching maximum number of about seven million. At this point, the ovary can produce and detect androgens and estrogen, though it is unknown whether human fetal ovaries in vivo produce androgens in response to insulin [56]. Intra-uterine androgen exposure has been reported to affect programming of adrenal and pituitary pathway in human and animal models [57,58].

In the rhesus monkey, exposure to androgen excess early in gestation led to PCOS phenotype with hyperandrogenism, oligomenorrhea, LH hypersecretion, enlarged multi-follicular ovaries in addition to insulin resistance, hyperlipidemia, and increased abdominal adiposity [59].

In a clinical study of pregnant women, testosterone levels at 18 weeks correlated with early follicular AMH levels in their daughters [60]. Examination of placental tissue in women with PCOS showed higher 3β-HSD-1 activity and lower P450 aromatase activity (pathogenic of hyperandrogenism) compared to control [61].

### 4.2. Environmental Exposures

Exposure to endocrine-disrupting chemicals in utero or at other critical developmental periods have been hypothesized to contribute to PCOS. A “two-hit” hypothesis of genetic and environmental exposures has been suggested. Levels of bisphenol A (BPA) are higher in adolescents [62] and women with PCOS than controls, even after controlling for obesity, and correlate to testosterone levels [63,64]. BPA levels were also negatively correlated with antral follicle count, a marker of ovarian reserve [65]. Another group reported higher levels of perfluorinated compounds and lower levels of phthalate metabolites in serum and urine of PCOS women than controls [66].

### 4.3. Gut Microbiome

As it is become clear that sex steroids affect the gut microbiome, recent studies of animal and human models have examined the potential role of the gut microbiome in PCOS [67]. A Letrozole-induced mouse model of PCOS found lower diversity and increase in Bacteroides and Firmicutes species that were previous associated with mouse models of metabolic disease [68]. A rat model using prenatal androgen exposure also exhibited altered gut microbial composition [69] along with cardiovascular risk factors. Human studies similarly found lower alpha diversity in PCOS with both alpha and beta diversity correlated to markers of hyperandrogenism [70,71]. Results from a small pilot study suggested that gut dysbiosis was more severe in PCOS subjects with insulin resistance [72]. Another study which compared obese and non-obese PCOS, control women and control men actually found reduced alpha diversity in all women compared to men, whereas beta diversity was reduced only in obese PCOS women; overall diversity and abundance of several specific general correlated positively to testosterone levels and negatively to estradiol [73]. Of note, there is no data on gut microbiome in pediatric PCOS, and it is unclear how early these changes occur.

### 4.4. Genetics

Great focus has been placed on trying to identify genetic mediators of PCOS. While this work has predominantly been done in adult PCOS populations rather than adolescents, we have summarized the major areas of genetic investigation. Genome wide association studies (GWAS) have been performed in various cohorts of women with PCOS and led to identification of many candidate genes implicated in diverse pathways, including insulin signaling, gonadotropin axis, ovarian folliculogenesis, and sex steroid production [74,75,76,77,78]. Ultimately, GWAS has yielded conflicting data with significant population variation and lack of reproducibility; no clear genetic etiology can be supported nor can any genotype predict PCOS risk in the general population. 

More recent studies are looking beyond genome to the epigenome, with analysis of DNA methylation. A number of such studies have found altered DNA methylation at loci identified originally via GWAS, including *LHCGR, INSR, YAP1*, and *TOX3* [79]. Genome-wide DNA methylation analysis of human ovarian granulosa cells revealed thousands of differentially methylated CpG sites in PCOS versus controls, interestingly, there were more sites identified in the lean PCOS group versus the obese group [80]. Other studies implicate hypomethylation at specific sites in ovarian and blood cells [81,82]. 

Alterations in levels of specific micro-RNAs, which regulate gene expression in myriad pathways have also been explored in recent studies [83] and found to affect expression of genes involved in androgen activity, glucose metabolism, and follicular development. Micro-RNA alterations have been described in ovarian theca and granulosa cells, adipose tissue, and follicular fluid in addition to serum [84,85,86].

## 5. No Magic Pill: Explorative Treatment Options

Standard treatment options for PCOS include lifestyle changes, the insulin sensitizer metformin, anti-androgens, and estrogen-progestin oral contraceptive pills with a focus on restoring menstrual regularity, reducing dermatologic complications, and improving metabolic health [3,55].

As these treatment options for PCOS are mostly symptomatic and have not been shown to alter the overall course and progression of disease, identification of novel therapeutic modalities remains a priority for clinicians and scientists in the sphere. 

### 5.1. Metabolic Modulation

Given the strong association of PCOS with insulin resistance, diabetes, and cardiometabolic disease, and the efficacy of the diabetes medication metformin, drugs designed for the treatment of diabetes are a main focus of therapeutic trials in PCOS. In an open-label study of obese and overweight women with PCOS, the glucagon-like peptide-1 (GLP-1) analog, exenatide, led to significant weight loss and improvements in menstrual cycling after 12 weeks [87]. In a cohort of overweight, insulin–resistant women with PCOS, metformin combined with exenatide improved weight loss, hormonal and metabolic parameters, and menstrual cyclicity better than either metformin or exenatide alone [88]. Another diabetes medication, the dipeptidyl peptidase-4 inhibitor saxagliptin also showed greater efficacy when combined with metformin versus either drug alone in a group of subjects with PCOS and pre-diabetes [89]. Two studies report that the β-Hydroxy β-methylglutaryl-CoA reductase inhibitor simvastatin, generally used for hyperlipidemia and protection from atherosclerotic heart disease, was found to be effective in improving ovarian morphology and cardiometabolic parameters as monotherapy [90] and in combination with metformin [91]. As statins have been linked to increased incidence of type 2 diabetes [92], further understanding of short and long-term effects on glucose metabolism in PCOS are required.

While most of these studies have been done in adult populations, one group has explored the effects of pioglitazone, a peroxisome proliferator-activated receptor gamma agonist that was previously a common treatment for diabetes, in adolescents with PCOS. Combination of spironolactone, pioglitazone, and metformin (i.e., SPIOMET) in low doses was compared to oral contraceptive treatment for 12 months resulted in higher ovulation rates, as well as loss of visceral fat and improved insulin sensitivity despite no change in body weight [93].

### 5.2. Supplements

While lifestyle changes and promoting healthy dietary intake remain the foundation of PCOS treatment, the use of specific nutritional and other supplements have been studied extensively in this population. 

A meta-analysis of omega-3 fatty acids in PCOS showed improvement in insulin resistance and, Homeostatic model assessment of insulin resistance (HOMA-IR), adiponectin, and expected decrease in triglyceride levels in women with PCOS, though no significant effects on androgen levels or ovulation rates were seen [94]. In contrast, an earlier meta-analysis including fewer studies, showed no difference in insulin or HOMA-IR [95].

Myo-inositol is a carbocyclic sugar found in many plants but also naturally formed in the human body from glucose; it functions as a cell signal mediator. It has been studied as a treatment for restoring hormonal and metabolic balance in PCOS, alone or in combination with its isomer D-chiro-inositol; improvements in hormonal function are proposed to be secondary to reduction in insulin resistance [96]. In fact, a metanalysis did show significant decreases in fasting insulin and HOMA-IR with trend to lower testosterone with myo-inositol supplementation [97]. In a study of adolescent girls with PCOS, myo-inositol alone led to reductions in weight, metabolic parameters, and free testosterone, whereas in combination with oral contraceptives, weight remained unchanged (compared to weight gain in OCP only group) and led to improvements in metabolic and hormonal profile [98]. At this time overall evidence is lacking to support clinical use of this therapy, but it deserves further study [3].

Vitamin D has been frequently explored for possible cardiometabolic benefits in diverse populations, including PCOS. Polymorphisms in the vitamin D receptor (VDR) gene have been found to be a risk factor for PCOS [99,100]. In one clinical trial, high dose vitamin D (4000 units daily) for 12 weeks in insulin resistant women with PCOS led to lower free androgen index compared to low dose vitamin D (1000 units) or placebo [101]. Another study by the same group, using 50,000 units vitamin D every other week for eight weeks in infertile women with PCOS showed lower AMH levels and improved insulin and lipid gene expression compared to placebo, as well as improved mental health scores [102]. In vitamin D deficient women with PCOS, high dose supplementation improved metabolic markers, including adiponectin, HOMA-B, and fasting glucose [103]. A study of low dose supplementation showed modest decreases in HOMA-IR but significant reduction in liver enzymes (a marker of fatty liver disease) [104].

The trace metal selenium has gained interest due to purported anti-oxidant and insulin-mimicking properties [105]. Supplementation with selenium combined with probiotics for 12 weeks was shown by one group to improve mental health parameters and androgen levels in women with PCOS [106]. In contrast, another study found that selenium supplementation worsened insulin resistance in PCOS [107].

Magnesium, zinc, and soy isoflavones have also been reported to have beneficial effects in women with PCOS, though all studies are short-term and have not yet been widely replicated [108,109].

## 6. Conclusions

PCOS is a common and complex condition with multi-faceted etiologic origins and represents a major long-term health risk for affected adolescents and women. Our understanding of the comorbidities, pathogenesis, and treatment options continues to evolve as the science advances. While progress has been made, there remain many unanswered questions and fertile areas for future scientific inquiry. 
